# The role of prognostic nutritional index in the management of pulmonary sarcomatoid carcinoma

**DOI:** 10.1186/s13569-020-00148-2

**Published:** 2020-12-07

**Authors:** Yan Wang, Yu Cao, Junfeng Liu

**Affiliations:** 1 Department of Thoracic Surgery, The Fourth Hospital of Hebei Medical University, No. 12, Jiankang Road, Shijiazhuang, 050000 China; 2 Department of medical statistics, The Fourth Hospital of Hebei Medical University, No. 12, Jiankang Road, Shijiazhuang, 050000 China

**Keywords:** Pulmonary sarcomatoid carcinoma, Prognostic nutritional index, Prognosis

## Abstract

**Background:**

Pulmonary sarcomatoid carcinoma is characterized by poor survival rates compared with other non-small cell lung cancer. Prognostic nutritional index has significant prognostic value in many malignant tumors. We conducted this retrospective study to investigate the role of prognostic nutritional index in patients with pulmonary sarcomatoid carcinoma and to determine prognostic factors.

**Methods:**

Of 8176 patients with resected lung cancer in a single high-volume institution between 2008 and 2015, 91 patients with pathologically diagnosed sarcomatoid carcinoma were included in our study and evaluated. Kaplan–Meier analysis and Cox regression analysis were conducted to analyze clinicopathologic data. Subgroup analysis of overall survival (OS) and recurrence-free survival (RFS) among pulmonary sarcomatoid carcinoma patients were also conducted.

**Results:**

Univariable analysis showed that tumor size (P = 0.018 in OS), and P = 0.021 in RFS), tumor stage(P < 0.001 in OS, and P = 0.002 in RFS), nodal metastasis (P < 0.001 in OS, and P < 0.001 in RFS), pathological stage (P < 0.001 in OS, and P < 0.001 in RFS), treatment modality (P = 0.032 in OS, and P = 0.059 in RFS) and PNI (P < 0.001 in OS, and P < 0.001 in RFS), were significant factors of both OS and RFS. In multivariable analysis, for OS, the pathological stage (Hazard ratio (HR) 1.432; 95% confidence interval (95% CI) 1.210–1.695; P < 0.001) and PNI (HR 0.812; 95% CI 0.761–0.865; P < 0.001) were independent prognostic factors. And for RFS, We found PNI as an independent prognostic factor (HR 0.792; 95% CI 0.739–0.848; P < 0.001), and the pathological stage (HR 1.373; 95% CI 1.160–1.625; P < 0.001). In the subgroup of patients with PNI ≥ 49.4, univariable analysis showed treatment modality was a significant factor of overall survival (P = 0.001); multivariable analysis showed patients received postoperative chemotherapy (HR 0.288; 95% CI 0.095–0.874; P = 0.028) or postoperative chemotherapy with targeted therapy (HR 0.148; 95% CI 0.030–0.726; P = 0.019) has better overall survival rates.

**Conclusion:**

The PNI and the pathological TNM stage are independent prognostic factors for pulmonary sarcomatoid carcinoma. PNI is an important indicator for the selection of postoperative adjuvant therapy. Patients with PNI ≥ 49.4 may benefit from postoperative chemotherapy and targeted therapy. We still need further prospective studies to confirm these results.

## Background

Pulmonary sarcomatoid carcinoma (PSC) is considered as a rare subtype of non-small cell lung cancers with very aggressive behavior [1]. The proportion of patients with pulmonary sarcomatoid carcinoma developed recurrence, even after R0 surgery is appreciable [2]. Studies reported poor survival outcome in patients with early-stage PSCs [3]. Several case report showed that PSC is resistant to chemotherapy [1, 3–5]. Therefore, predicting the prognosis of PSC patients accurately is important to improve PSC patients’ survival and to provide important information to the management of PSC patients.

The postoperative complications, and the long-term outcomes of patients with malignances have been considered to be associated significantly with preoperative nutritional condition and immunological status [6–8]. The prognostic nutritional index (PNI), calculated based on combining the serum albumin concentration with total peripheral lymphocyte count, was initially used to assess the immune-nutritional status before or after surgery and postoperational complications in patients underwent gastrointestinal surgery [7]. Recent study show that the PNI is a prognostic factor for various carcinomas [8–10]. PNI has not yet been investigated in PSC patients to our knowledge. Therefore, we studied the correlation between the PNI and clinical characteristics and the PNI’s impact on the overall survival (OS) and recurrence-free survival (RFS) in PSC patients.

## Methods

### Patients

Medical records between January 2008 and December 2015 were reviewed for 8176 consecutive patients with resected lung cancer and lymph node dissection at the 4th hospital of Hebei Medical University. Patients diagnosed as pulmonary sarcomatoid carcinoma with R0 resection and complete clinic-pathological data were included for analysis. The laboratory results were obtained within 1 week before operation. Owing to the retrospective design, patient consent was waived. The study protocol was approved by the Ethics Committee of the 4th hospital of Hebei Medical University. The follow-up was conducted at clinic and by telephone call from designated personale in follow-up center in our hospital until October 31, 2019, or patient’s death. We obtained the clinical characteristics of patients retrospectively from medical records and evaluated these characteristics as prognostic factors. These factors included the patient’s age, sex, smoking and drinking habits, tumor size, tumor stage, tumor location, lymph node metastasis, therapeutic methods, and pathological stage. Preoperative data were obtained from the patients’ medical records, including serum albumin and total lymphocyte count from peripheral blood tests. Then, the following formula was used to calculate the PNI: 10* serum albumin (g/dl) + 0.005* total lymphocyte count (cells per mm^3^) [7].

### Statistical analysis

The categorical variables between groups were compared using the X^2^ test. Continuous variables with normal distribution were compared using the t test. The median value of the follow-up was 51 months. The OS was considered as the time from the operation to death or last follow-up. The survival curves were generated by the Kaplan–Meier method. Differences among the curves were evaluated by log-rank test. The mean of PNI was used as the cutoff value since PNI value confirm to the normal distribution in the population. According to the cutoff value of the PNI, all patients were divided into a PNI-high group or a PNI-low group. Variables found significant in the univariable analysis were entered into a multivariable analysis (COX proportional hazards models). We also try to consider PNI as a continuous variable in the time-to-event models when studying the independent association of PNI with OS and RFS. All P values of < 0.05 were considered to be significant, and confidence intervals (CI) were calculated at the 95% level. The statistical analyses were performed using SPSS software (version 24.0; SPSS, Chicago, IL).

## Results

### Data

None of these patients received preoperative chemotherapy. Postoperative adjuvant chemotherapy with an platinum-based regimen (pemetrexed + cisplatin) was accepted in a total of 30 patients. And 30 patients received postoperative adjuvant chemotherapy with targeted therapy. We used the 8th edition of the American Joint Committee on Cancer TNM classification system to classified the stage of PSC [11].

### PNI and characteristics of patients

At time of final follow-up, we totally monitored 91 patients with completely resected PSC for a median of 51 months (range 2–89). In the PNI < 49.4 group the follow-up time: 1–67 (months), and in the PNI ≥ 49.4 group follow-up time: 39–89 (months). An expert pathologist performed the pathologic revision of the samples in a centralized blind way. During the revision, spindle cell carcinoma, giant cell carcinoma, pleomorphic carcinoma, carcinosarcoma, and pulmonary blastoma as 5 different subtypes of PSC were identified and distinguished. Spindle and giant cell carcinoma represented 95.6%, pleomorphic carcinoma 2.2%, carcinosarcoma 1.1% and pulmonary blastoma 1.1% of our whole cohort.

Mean preoperative PNI was 49.4, and standard deviations was 5.6 (range 41.1–59.2). PNI correlated significantly with tumor stage, lymph node metastasis, TNM stage and treatment modality (Table 1). We found patients with T3–4 has significantly lower PNI than patients with T1–2 (48.30 vs. 51.53, P = 0.008). Patients with N0 have higher PNI than patients with N1–3 (50.35 vs. 48.01, P = 0.048). Patients with stage Ia–IIa have significantly higher PNI compared with patients with more advanced TNM stage (51.96 vs. 47.88, P = 0.001). Patients simply underwent surgery have significantly lower PNI than patients with postoperative adjuvant therapy (48.39 vs. 51.47, P = 0.012). The association between PNI and sex, age, smoking history, alcohol abuse history, tumor size, tumor location, or histologic subtype was not significant (P > 0.05). A PNI mean of 49.4 was applied to divide patients in this study. We subsequently stratified all patients into two groups, high PNI group (PNI ≥ 49.4; n = 39) and low PNI group (PNI < 49.4; n = 52). We didn’t find the distributions of TNM categories differ significantly between these two groups.

**Table 1:** PNI and clinicopathological characteristics relationship

Variables	Cases (n)	PNI (mean ± SD)	P-value
Sex			0.084
Male	69	48.83 ± 5.89	
Female	22	51.19 ± 4.07	
Age (years)			0.170
≤ 60	51	50.11 ± 5.66	
> 60	40	48.49 ± 5.39	
Smoking history			0.692
Former/current smoker	51	49.19 ± 5.98	
Never-smoker	40	49.66 ± 5.07	
Alcohol abuse			0.615
Yes	37	49.04 ± 6.05	
No	54	49.65 ± 5.26	
Histologic subtype			0.919
Giant/spindle cell carcinoma	87	49.41 ± 5.60	
Other subtype	4	49.12 ± 5.81	
Tumor size (cm)			0.120
≤ 5	38	50.48 ± 5.82	
> 5	53	48.63 ± 5.31	
Tumor stage			0.008
T1–2	31	51.53 ± 5.23	
T3–4	60	48.30 ± 5.46	
Tumor location			0.670
Peripheral	37	48.76 ± 6.04	
Central	9	49.78 ± 4.42	
Both	45	49.85 ± 5.43	
Nodal metastasis			0.048
N0	54	50.35 ± 5.68	
N1–3	37	48.01 ± 5.17	
Pathological stage(8th edition)			0.001
Ia–IIa	34	51.96 ± 5.31	
IIb–IIIc	57	47.88 ± 5.19	
Treatment modality			0.959
Surgery	31	47.97 ± 5.28	
Surgery + adjuvant CT	30	51.27 ± 5.46	
Surgery + adjuvant CT + targeted therapy	30	49.00 ± 5.63	

PNI: prognostic nutrition index, SD: Standard deviation. CT: chemotherapy

### PNI and survival

To evaluate the importance of PNI in survival, we try to consider PNI as a continuous variable in the time-to-event models. We compared OS and RFS in patients categorized by gender (males versus females), age (younger versus older than 60 years), smoking status (former or current smokers versus never-smokers), alcohol status (alcohol abuser versus none alcohol abuser), tumor size (tumor maximum diameter equal or less than 5 cm versus larger than 5 cm), tumor stage (T1–2 versus T3–4), tumor location (peripheral vs. central), nodal metastasis (N0 vs. N1–3), pathological stage (Ia–IIa vs. IIb–IIIc), treatment modality (patients received adjuvant therapy vs. patients did not received adjuvant therapy), and PNI. Univariable analysis showed that tumor size (P = 0.018 in OS, and P = 0.021 in RFS), tumor stage (P = 0.001 in OS, and P = 0.002 in RFS), nodal metastasis (P < 0.001 in OS, and P < 0.001 in RFS), pathological stage (P < 0.001 in OS, and P < 0.001 in RFS), treatment modality (P = 0.310 in OS, and P = 0.365 in RFS) and PNI (P < 0.001 in OS, and P < 0.001 in RFS), were significant factors of both OS and RFS. Tumor stage and nodal stage are the components of pathological stage and cannot be used as independent factors in multivariable analysis. In multivariable analysis, for OS, we found the pathological stage (HR 1.432; 95% CI 1.210–1.695; P < 0.001) and PNI (HR 0.812; 95% CI 0.761–0.865; P < 0.001) were independent prognostic factors. And for RFS, we found PNI (HR 0.792; 95% CI 0.739–0.848; P < 0.001) and the pathological stage (HR 1.373; 95% CI 1.160–1.625; P < 0.001) were independent prognostic factors (Table 2). With one-unit increase in PNI, we can see a 18.8% drop in the instantaneous risk of death, and a 20.8% drop in the instantaneous risk of recurrence.

**Table 2:** Uni and multivariable survival analysis for various potential prognostic factors of overall and recurrence-free survival in PSC patients

OS	Univariate analysis			Multivariate analysis			RFS	Univariate analysis		Multivariate analysis		
	HR	95% CI	P value	HR	95% CI	P value	HR	95% CI	P value	HR	95% CI	P value
Age <= 60 vs. > 60	1.263	0.795–2.008	0.323				1.279	0.805–2.033	0.297			
Sex Male vs. Female	0.778	0.445–1.358	0.377				0.765	0.438–1.335	0.345			
Smoking history Yes vs. No	1.033	0.647–1.649	0.892				1.048	0.657–1.674	0.843			
Alcohol abuse Yes vs. No	0.899	0.560–1.445	0.660				0.913	0.568–1.468	0.707			
Tumor size > 5 vs. <=5 cm	1.811	1.109–2.956	0.018				1.776	1.090–2.896	0.021			
Tumor stage T3–4 vs. T1–2	1.884	1.291–2.750	0.001				1.807	1.247–2.620	0.002			
Tumor location Peripheral vs. Central	1.044	0.741–1.470	0.805				1.035	0.735–1.459	0.843			
Nodal metastasis Positive vs. Negative	1.771	1.350–2.324	< 0.001				1.809	1.372–2.386	< 0.001			
Pathological stage IIb–IIIc vs. Ia–IIa	1.546	1.313–1.820	< 0.001	1.432	1.210–1.695	< 0.001	1.539	1.304–1.817	< 0.001	1.373	1.160–1.625	< 0.001
Treatment modality			0.032			0.310			0.059			0.365
S + CT/S	0.473	0.268–0.835	0.010	0.639	0.356–1.145	0.132	0.508	0.289–0.893	0.019	0.673	0.379–1.197	0.178
S + CT + Targeted therapy/S	0.634	0.360–1.118	0.115	0.883	0.499–1.561	0.669	0.679	0.386–1.194	0.179	0.951	0.538–1.680	0.862
PNI	0.799	0.753–0.849	< 0.001	0.812	0.761–0.865	< 0.001	0.781	0.733–0.833	< 0.001	0.792	0.739–0.848	< 0.001

HR: Hazard ratio; CI: confidence interval

### PNI and long-term outcome

**Fig. 1:**
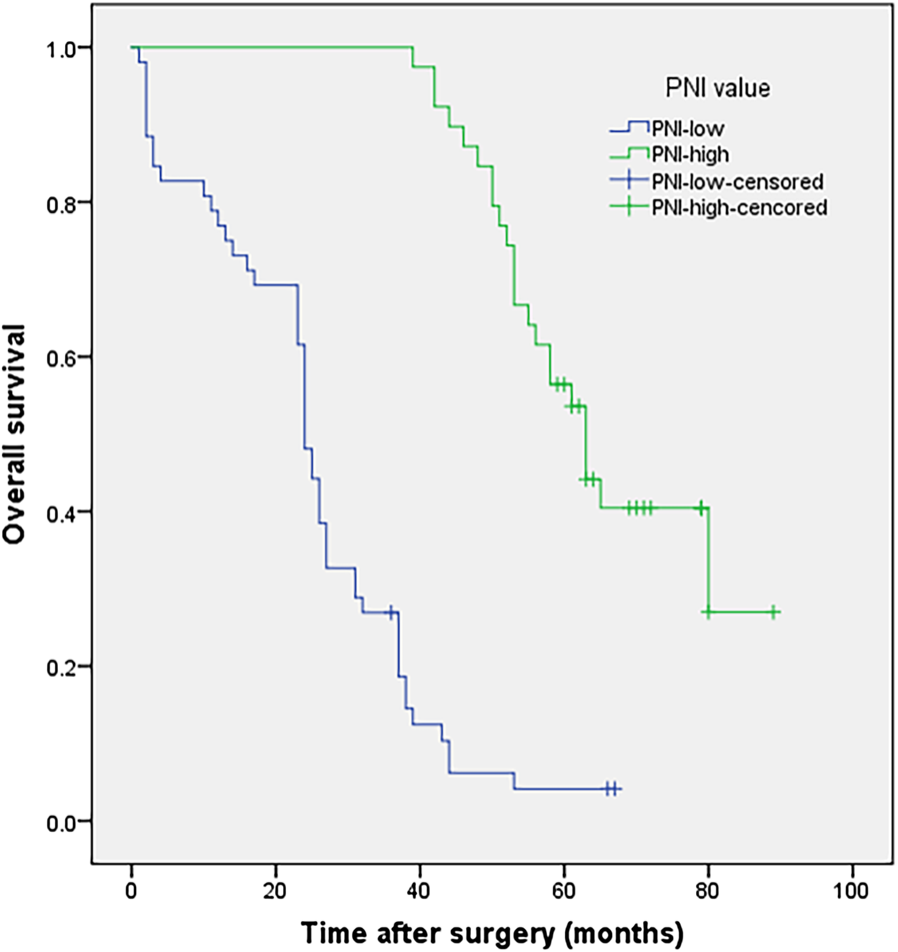
Kaplan–Meier survival curves evaluate the overall survival (OS) in patients with completely resected PSC (n = 91) stratified according to prognostic nutritional index (PNI). OS rate was significantly worse for patients with PNI less than 49.4 than for patients with PNI equals or higher than 49.4 (P < 0.001)

**Fig. 2:**
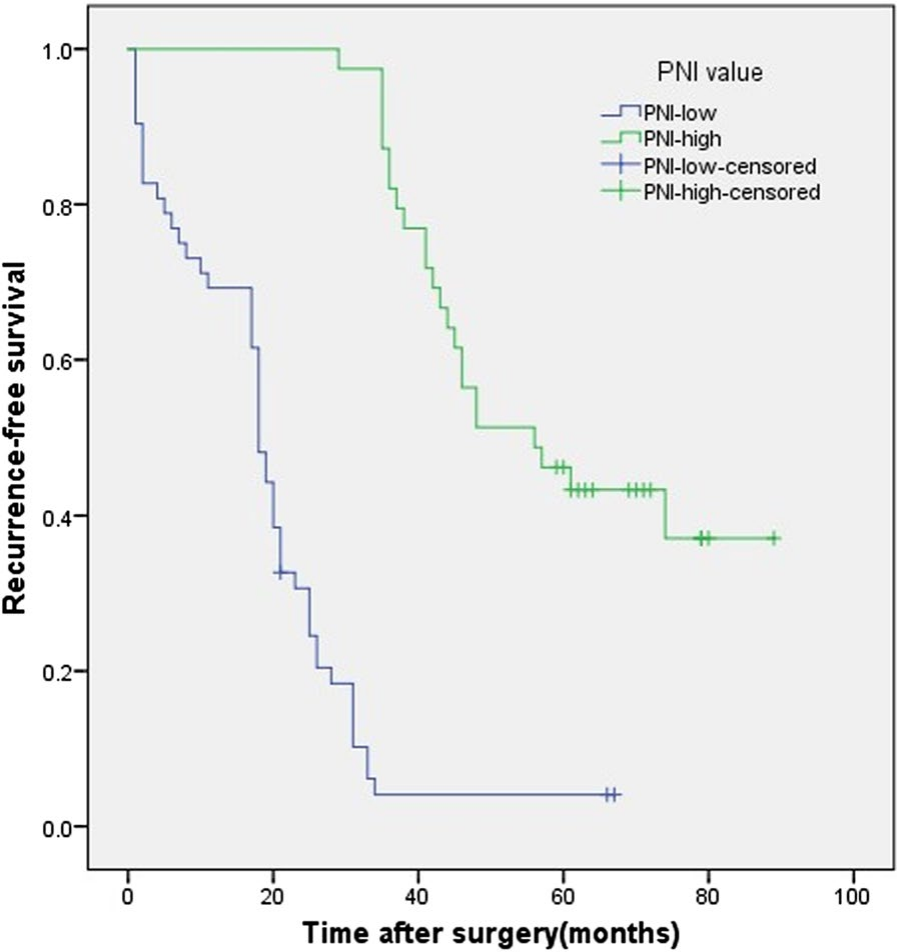
Kaplan–Meier survival curves evaluate the recurrence-free survival (RFS) in patients with completely resected PSC (n = 91) stratified according to prognostic nutritional index (PNI). RFS rate was significantly worse for patients with PNI less than 49.4 than for patients with PNI equals or higher than 49.4 (P < 0.001)

**Fig. 3:**
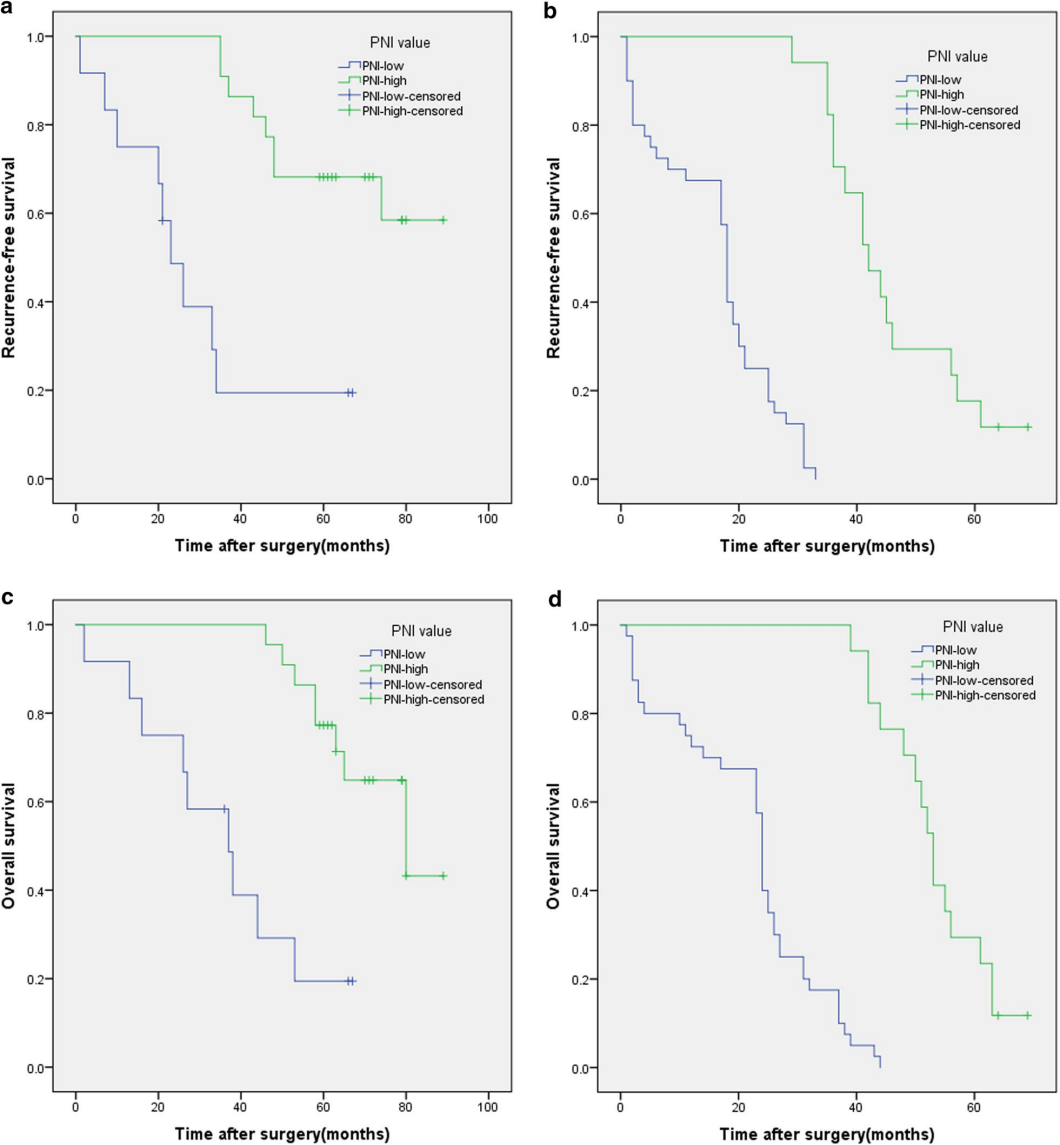
Kaplan–Meier survival curves evaluate the overall survival (OS) and recurrence-free survival (RFS) in patients with completely resected PSC (n = 91) stratified according to prognostic nutritional index (PNI) among patients with stage pIa–IIa (**a** OS; **b** RFS) or pIIb–IIIc (**c** OS; **d** RFS) disease. Hazard ratios (HR) of PNI for OS and RFS are lower in stage pIIb–IIIc than in stage pIa–IIa (**a** HR 0.167, 95% CI 0.060–0.463; **b** HR 0.145, 95% CI 0.052–0.410; **c** HR 0.052, 95% CI 0.017–0.156; **d** HR 0.017, 95% CI 0.002–0.129. CI: confidence interval)

In Kaplan–Meier analysis, 5-year OS rates were 4.1% and 56.4% in the low- and high-PNI groups (≥ 49.4 vs. < 49.4), respectively (Fig. 1) (P < 0.001). Five-year RFS rates were 4.1% and 46.2% in the low- and high-PNI groups, respectively (Fig. 2) (P < 0.001). Figure 3a–d showed 5-year OS and RFS curves which were stratified according to PNI among patients with stage pIa–IIa and pIIb–IIIc disease. In the group of patients with stage pIa–IIa disease, HR for OS was 0.167 (95% CI 0.060–0.463) and HR for RFS was 0.145 (95% CI 0.052–0.410) and in the group of patients with stage pIIb–IIIc disease, HR for OS was 0.052 (95% CI 0.017–0.156) and HR for RFS was 0.017 (95% CI 0.002–0.129).

### PNI and causes of death

During follow-up, 23 patients (59.0%) and 49 patients (94.2%) in the high- and low-PNI group died, respectively. In the high-PNI group, causes of death included tumor recurrence (n = 10 [43.5%]), other malignancies (n = 8 [34.8%]), and other causes (n = 5 [21.7%]), respectively. And in the low-PNI group, tumor recurrence (n = 41 [83.7%]), other malignancies (n = 5 [10.2%]), and other causes (n = 3 [6.1%]), respectively. Significant difference was found between the low-PNI group (78.8%) and the high-PNI group (25.6%; P = 0.005) for tumor recurrence–related death.

### PNI and treatment therapy

**Fig. 4:**
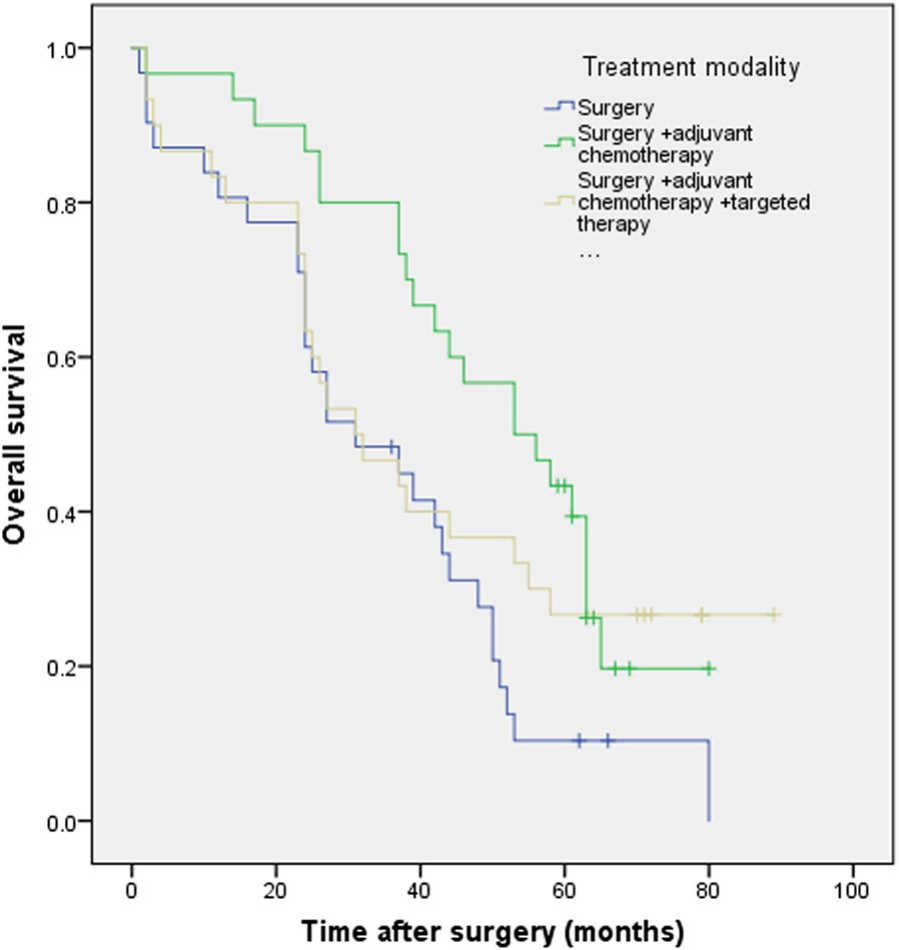
Kaplan–Meier survival curves evaluate the overall survival (OS) in patients with completely resected PSC (n = 91) stratified according to treatment modality. The difference in OS rates was significant between the group of patients with surgery and the group of patients with postoperative chemotherapy (P = 0.004) but not with postoperative chemotherapy and targeted therapy (P = 0.185), neither between the group of patients with postoperative chemotherapy and the group of patients with postoperative chemotherapy and targeted therapy (P = 0.331)

The difference in OS rates was significant between the group of patients with surgery and the group of patients with postoperative chemotherapy (P = 0.004) but not with postoperative chemotherapy and targeted therapy (P = 0.185), neither between the group of patients with postoperative chemotherapy and the group of patients with postoperative chemotherapy and targeted therapy (P = 0.331) (Fig. 4).

**Fig. 5:**
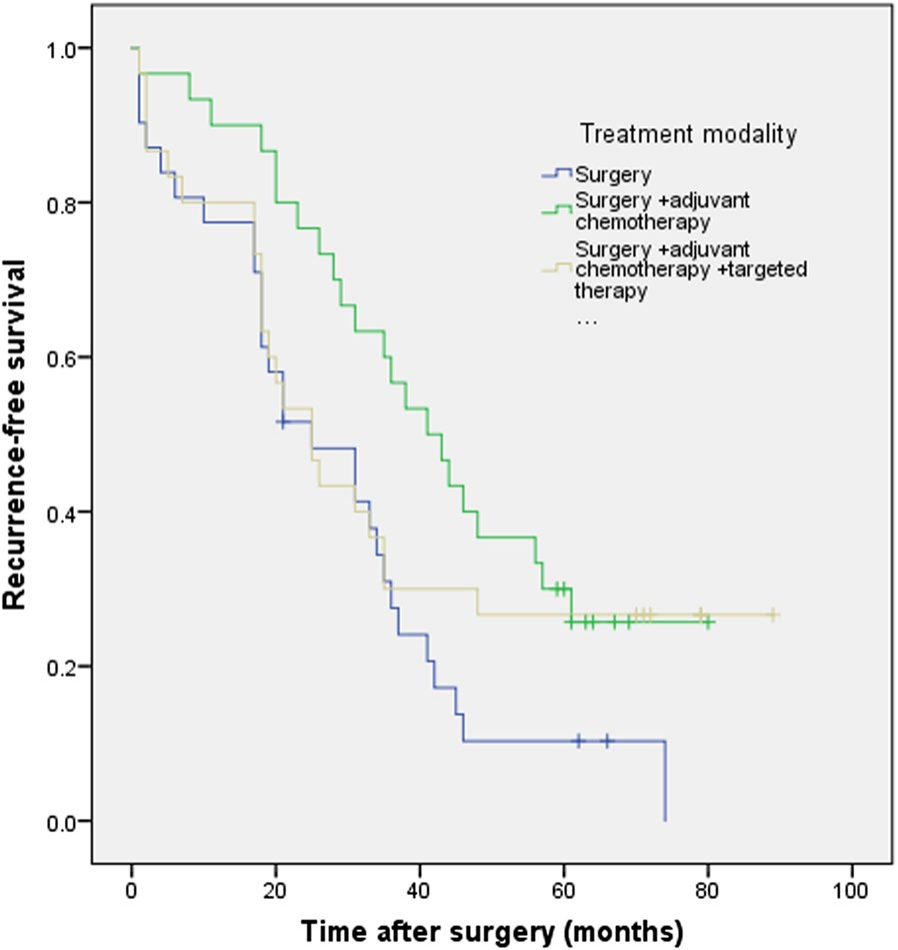
Kaplan–Meier survival curves evaluate the recurrence-free survival (RFS) in patients with completely resected PSC (n = 91) stratified according to treatment modality. The difference in RFS rates was significant between the group of patients with surgery and the group of patients with postoperative chemotherapy (P = 0.009) but not with postoperative chemotherapy and targeted therapy (P = 0.229), neither between the group of patients with postoperative chemotherapy and the group of patients with postoperative chemotherapy and targeted therapy (P = 0.295)

The difference in RFS rates was significant between the group of patients with surgery and the group of patients with postoperative chemotherapy (P = 0.009) but not with postoperative chemotherapy and targeted therapy (P = 0.229), neither between the group of patients with postoperative chemotherapy and the group of patients with postoperative chemotherapy and targeted therapy (P = 0.295) (Fig. 5).

**Fig. 6:**
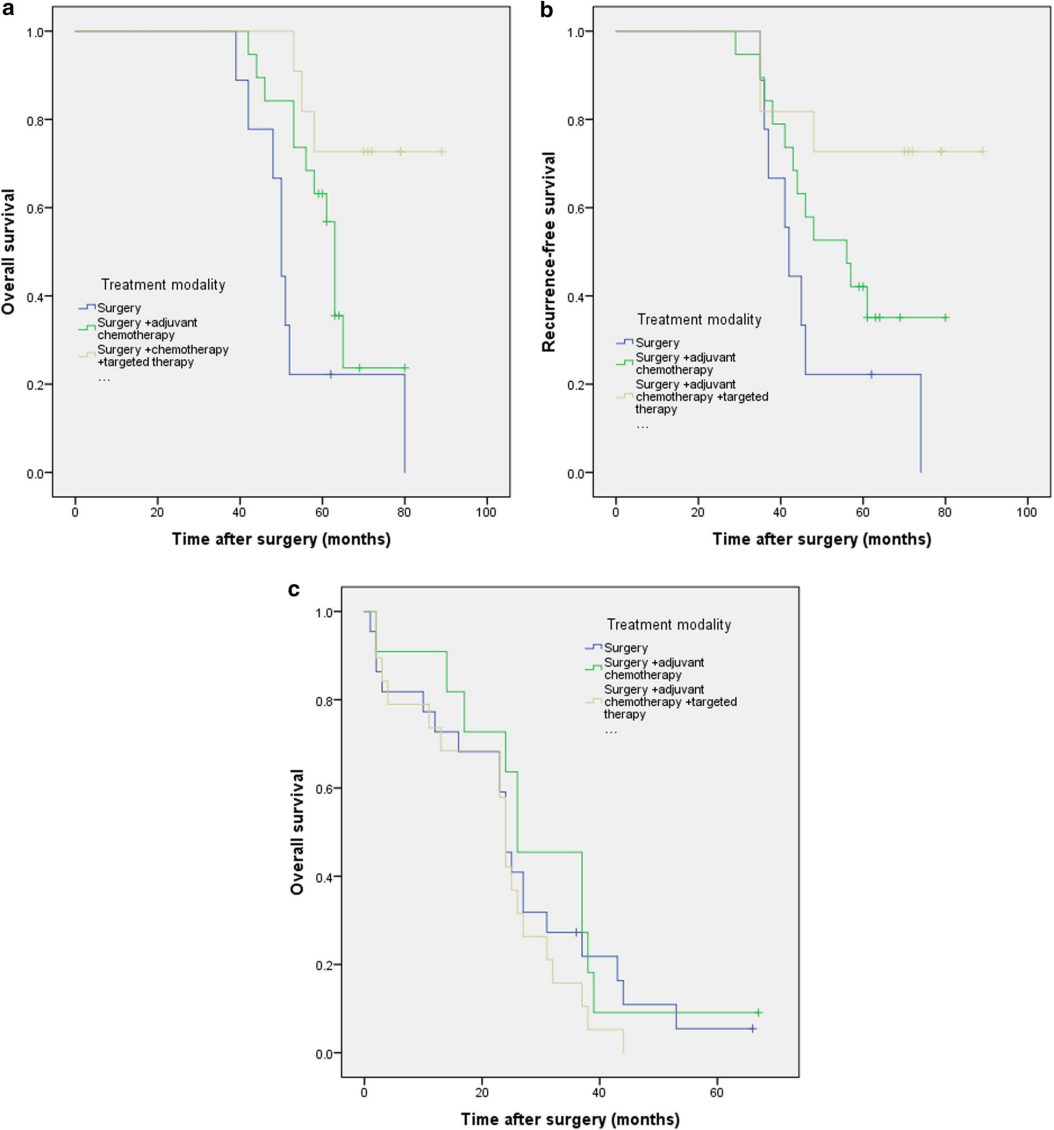
Kaplan–Meier survival curves evaluate the overall survival (OS) and recurrence-free survival (RFS) in patients with completely resected PSC (n = 91) stratified according to treatment modality among patients with higher PNI (PNI ≥ 49.4) (**a** OS, P = 0.002; **b** RFS, P = 0.020) or lower PNI (PNI < 49.4) (**c** OS, P = 0.369; **d** RFS, P = 0.466)

In the subgroup of patients with PNI ≥ 49.4, univariable analysis showed treatment modality was a significant factor of overall survival (P = 0.001) and recurrence-free survival (P = 0.005); multivariable analysis showed patients received postoperative chemotherapy (HR 0.288; 95% CI 0.095–0.874; P = 0.028) or postoperative chemotherapy with targeted therapy (HR 0.148; 95% CI 0.030–0.726; P = 0.019) has better overall survival rates; In the subgroup of patients with PNI < 49.4, we didn’t find the same results. In the subgroup of patients with PNI ≥ 49.4, patients received postoperative chemotherapy with targeted therapy has better OS rates than those received postoperative chemotherapy (P = 0.048, X^2^ = 3.924); In the subgroup of patients with PNI < 49.4, we didn’t find the same results (P = 0.143, X^2^ = 2.145) (Tables 3, 4) (Fig. 6a–d).

**Table 3:** Uni and multivariable survival analysis for various potential prognostic factors of overall and recurrence-free survival in PSC patients with PNI more than 49.4

OS	Univariate analysis		Multivariate analysis			RFS	Univariate analysis		Multivariate analysis		
	P value	X^2^	HR	95% CI	P value		P value	X^2^	HR	95% CI	P value
Age <= 60 vs. > 60	0.585	0.298					0.564	0.333			
Sex Male vs. Female	0.897	0.017					0.949	0.004			
Smoking history Yes vs. No	0.963	0.002					0.942	0.005			
Alcohol abuse Yes vs. No	0.551	0.356					0.623	0.242			
Tumor size > 5 vs. <= 5 cm	0.008	7.045	1.311	0.442-3.886	0.625		0.012	6.373	1.770	0.585–5.349	0.312
Tumor stage T3–4 vs. T1–2	0.062	3.496					0.091	2.860			
Tumor location Peripheral vs. Central	0.194	1.687					0.157	2.001			
Nodal metastasis Positive vs. Negative	< 0.001	14.076	1.164	0.451–3.004	0.753		< 0.001	27.165	3.328	1.105–10.022	0.033
Pathological stage IIb–IIIc vs. Ia–IIa	< 0.001	15.921	1.658	0.981–2.805	0.059		<0.001	17.590	1.230	0.723–2.095	0.445
Treatment modality	0.001	11.621			0.035		0.005	7.783			0.487
S + CT/S			0.288	0.095–0.874	0.028				0.649	0.248–1.700	0.379
S + CT + Targeted therapy/S			0.148	0.030–0.726	0.019				0.407	0.86–1.923	0.257

**Table 4:** Uni and multivariable survival analysis for various potential prognostic factors of overall and recurrence-free survival in PSC patients with PNI less than 49.4

OS	Univariate analysis		Multivariate analysis			RFS	Univariate analysis		Multivariate analysis		
	P value	X^2^	HR	95% CI	P value		P value	X^2^	HR	95% CI	P value
Age <= 60 vs. > 60	0.573	0.317					0.487	0.483			
Sex Male vs. Female	0.899	0.016					0.953	0.004			
Smoking history Yes vs. No	0.951	0.004					0.941	0.005			
Alcohol abuse Yes vs. No	0.433	0.615					0.436	0.607			
Tumor size >5 vs. <=5 cm	0.839	0.041					0.869	0.027			
Tumor stage T3-4 vs. T1-2	0.013	6.226	1.556	0.849–2.857	0.153		0.018	5.598	1.647	0.871–3.116	0.125
Tumor location Peripheral vs. Central	0.298	1.084					0.308	1.041			
Nodal metastasis Positive vs. Negative	0.302	1.065					0.618	0.249			
Pathological stage IIb–IIIc vs. Ia–IIa	0.019	5.518	1.117	0.885–1.409	0.351		0.061	3.504	1.037	0.819–1.314	0.764
Treatment modality	0.353	0.861					0.366	0.816			
S + CT/S											
S + CT +Targeted therapy/S

## Discussion

This study offers the first evidence of the prognostic value of PNI in patients with completely resected pulmonary sarcomatoid carcinoma. Pulmonary sarcomatoid carcinoma (PSC) comprises less than 1% of lung cancers, and they respond poorly to systemic therapy [12]. Immuno-nutritional status plays an important role in postoperative outcomes. Therefore, we attempted to explore the PNI which was designed to represent immune-nutritional status as a new prognostic factor [8–11]. In our study, multivariable analysis showed that PNI and TNM classification are strong predicting factors in patients with resectable PSC.

In 2017, Lococo et al. in a multicenter study found that among their cohort, spindle cell carcinoma represented (29%), giant cell carcinoma (8%), pleomorphic carcinoma (62%), carcinosarcoma (1%), pulmonary blastoma (0%) [13]. In 2017, Rahouma et al. went through the the SEER database between 1973 and 2013, and revealed predominance of less aggressive histological subtypes in the 1993–2013 time period. They found spindle and giant cell carcinoma represented 72.9%, pleomorphic carcinoma 13.4%, carcinosarcoma 11.5% and pulmonary blastoma 2.2% [14]. Our findings in histologic subtype of PSCs seem to be inconsistent with these results of previous studies. In the present study, our results indicated that giant and spindle cell carcinoma represented the majority of the entire cohort. It might be reasonable that this rate was higher than those reported previously considering the race and region differences.

In our study, low PNI significantly correlated with advanced TNM stage, backing up the hypothesis that patients with a low PNI have a worse tumor progression. The advanced pathological stage may cause impaired immune-nutritional status. In this study, we didn’t find low PNI was associated with smoking which indicated that in the low PNI group, smoking-related inflammation’s contribution was limited. Similar results can be found in previous studies regarding resectable NSCLC [15]. We also found that low PNI significantly correlated with treatment modality. It might be reasonable that the patients with better nutrition status and less systemic inflammation may be recommended to receive adjuvant therapy at the pre-gene therapy time.

The most commonly used PNI, was first reported in prediction of postoperative complications in gastrointestinal operation [6]. Many studies have reported that the PNI has prognostic value for various malignancies [7–10]. Qiu et al. reported that the PNI was an independent prognostic factor for patients who were received radical surgery with NSCLC [16]. Hong et al. proved that PNI could assist to identify small cell lung cancer patients with poor prognosis [17]. Shoji et al. indicated the PNI’s value in predicting postoperative recurrence in patients with stage I NSCLC [18]. Similarly in this study, the hazard of the high-PNI group was lower than the one of the low-PNI group, which suggested that patients with lower PNI had reduced survival as a group, the PNI was an independent predicting factor in patients with resectable PSC. We also revealed that the incidences of recurrence and recurrence-related death were significantly higher in the low-PNI group. These results indicated that a low PNI may be strongly associated with disease-specific death, and lead to a worse outcome in patients with PSC.

Vieira et al. in 2016, Velcheti et al. in 2013, Fallet et al. in 2015 and Schrock et al. in 2017 presented the evidence of the spectrum of genomic abnormalities that PSCs harbor might be therapeutically actionable [19–22]. In 2017, Schrock et al. reported clinical outcomes for 10 PSC patients received targeted or immunotherapy, three had partial responses and three had stable disease [22]. These reports suggest that targeted therapies and immunotherapy might have encouraging outcomes for patients with PSCs. However, in this study, we found that the overall survival of patients who received adjuvant chemotherapy combined with targeted therapy was not significantly better than that of patients who simply received adjuvant chemotherapy. In this study, the adjuvant chemotherapy regimen was pemetrexed plus cisplatin for 4 cycles. After the emergence of targeted therapy, we prescribed gefitinib or icotinib orally for 2 years or until disease progression in patients with 19Del or L858R mutations along with adjuvant chemotherapy. According to the results of this study, patients with pulmonary sarcomatoid carcinoma who received adjuvant chemotherapy combined with targeted therapy do not have an improved prognosis compared with those who simply received adjuvant chemotherapy. Further stratified analysis revealed that the subgroup of patients with higher PNI (PNI ≥ 49.4) has better overall survival in the targeted therapy combined with adjuvant chemotherapy group than in the adjuvant chemotherapy group. The same advantage was not found in the subgroup of patients with PNI < 49.4. Therefore, we infer that the level of PNI in patients with pulmonary sarcomatoid carcinoma may determine whether they can benefit from targeted therapy after surgery. This study’s result suggested that targeted therapy combined with adjuvant chemotherapy could be a suitable option for patients with lung sarcomatoid carcinoma with higher PNI after surgery. For patients with pulmonary sarcomatoid carcinoma with PNI < 49.4, postoperative adjuvant chemotherapy could be a suitable choice.

The limitations of this study are: it is an observational study. Although it has standardized entry criteria, and predefined endpoints for assessing survival, it is difficult to draw a very strong conclusion on the basis of our retrospective study, because of the heterogeneity of real-life patient populations and the small sample size from one center. And that might be the reason that our study is at variance with the results from other observational surveys. Hence, a large-sample, double-blinded, randomized prospective study with multicenter-participated is warranted to validate our results.

## Conclusions

This study indicated that the PNI and the pathological stage system are strong predictors of OS and RFS for patients with PSC. Patients with low PNI have even worse prognosis in this population. PNI is an important indicator for the selection of postoperative adjuvant therapy. Patients with PNI ≥ 49.4 may benefit from postoperative chemotherapy and targeted therapy. We still need further prospective studies to confirm these results.

## Data Availability

All data and materials are kept in the library of the fourth hospital of Hebei Medical University.

## Abbreviations

PSC: Pulmonary sarcomatoid carcinoma
PNI: Prognostic nutritional index
OS: Overall survival
RFS: Recurrence-free survival
HR: Hazard ratio
CI: Confidence interval
